# Redescription of *Pseudopheraheveli* Kramer (Hemiptera, Cicadellidae) with the first description of the female

**DOI:** 10.3897/zookeys.976.54582

**Published:** 2020-10-20

**Authors:** Stuart H. McKamey

**Affiliations:** 1 Systematic Entomology Laboratory, PSI, Agricultural Research Service, U.S. Department of Agriculture, c/o National Museum of Natural History, P.O. Box 37012, Washington, D.C. 20013, USA U.S. Department of Agriculture Washington United States of America

**Keywords:** Auchenorrhyncha, Neotropical, new species, Proconiini, sharpshooter

## Abstract

*Pseudopheraheveli* Kramer is redescribed from Monteverde, Costa Rica. The female is described for the first time. Fifteen images of the species are provided, including genitalia.

## Introduction

Sharpshooters are members of the cosmopolitan Cicadellinae, the third largest leafhopper subfamily, with over 2,500 valid species among 357 genera. Sixty-three of these genera, and 468 species, belong to the New World tribe Proconiini (Young 1968; Marucci et al. 2002; Godoy 2005; Rakitov and Godoy 2005; McKamey 2007), which includes the genus *Pseudophera* Melichar.

Species of the genus *Pseudophera* are among the largest leafhoppers, ranging in length from 16 to 20.5 mm. Young (1968) revised the genus and listed six valid species, including one new species and a new synonym. He reported the distribution of the genus as Mexico (one species), Central America (four species), and one species each in Colombia, Ecuador, and Suriname. Subsequently, Kramer (1976) described *P.heveli* from Costa Rica, Nielson and Godoy (1995) described *P.chelicerata* and *P.jimenezi* from Costa Rica, and Emmrich (1999) described *P.paraensis* from Brazil and *P.zelayaensis* from Nicaragua, bringing the total number of species to eleven. As Young (1968) noted, *Pseudophera* is “distinguished by its large size and by its earlike, thick, rounded lobe on the epimeron of the metathorax” (Figs 2, 5). McKamey (2007) listed all species in the genus and Wilson et al. (2009) provided habitus images of 10 of them (not *P.zelayaensis*), including a female of *P.heveli* in the California Academy of Sciences, San Francisco, California, also from Monteverde, Puntarenas Province, Costa Rica. The new specimens, all from the type locality, represent both genders but were collected three years apart.

## Materials and methods

In providing distribution data, quotation marks separate labels and a vertical line separates lines on a label. All examined specimens are deposited in the United States National Museum of Natural History, Smithsonian Institution, Washington, DC (USNM).

Terminology for general morphology was based on Young (1968) and Dietrich (2005), while leg chaetotaxy follows Rakitov (1998).

A Leica MZ12 stereomicroscope was used to examine structures. The body length was measured using a digital micrometer. A manual 5 mm micrometer was used to determine ratios between other, shorter distances.

The abdomen was detached, macerated in a warmed 10% KOH solution for 24 hours at room temperature, bathed in water, then acetic acid to stop the reaction. After dissection, structures were stored in a glass microvial containing glycerin and pinned beneath the specimen.

**Figures 1–5.**
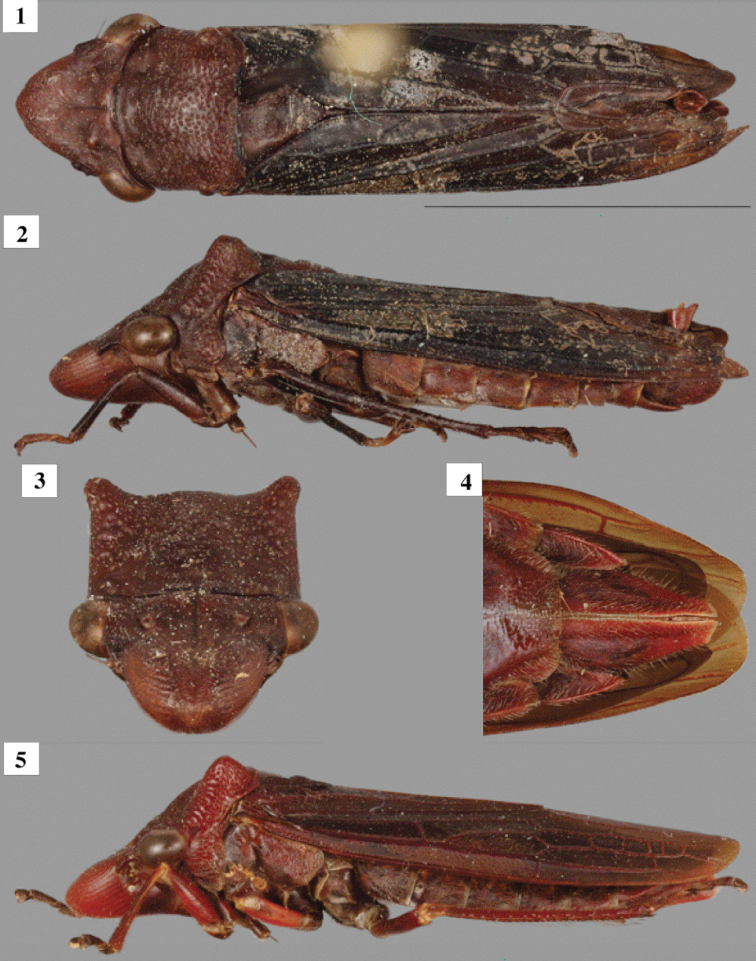
*Pseudopheraheveli*. Male (**1–3**) and female (**4, 5**) **1–3** habitus in dorsal, lateral, and anterior views, respectively **4** detail of undissected female sternum, ventral view **5** habitus, lateral view. Scale bar: 8 mm (**1**).

Images were taken with a Canon 5Dsr camera with an adjustable 65mm lens. Photos were taken using Capture One Pro version 10.1.2, 64 Bit, Build 10.1.2.23 imaging software, aided by CamLift version 2.9.7.1. The specimen was lit using two adjustable Dynalite MH2050 RoadMax flash heads, each attached to a Manfrotto 244 arm. The light was diffused using a simple, lampshade-style cone of translucent paper between the specimen and light sources. After individual “slices” were photographed, they were compiled into a single, composite image using Zerene Stacker – USDA SI-SEL Lab Bk imaging system, version 1.04, Build T201706041920. Stacked images were enhanced and edited in Adobe Photoshop CSS Extended version 12.0. The scale bar (in Fig. 1) was generated through Photoshop directly from the metadata of the photo.

## Results

### Pseudophera heveli

Taxon classificationAnimaliaHemipteraCicadellidae

Kramer, 1976

F8BED1E9-EF3E-50AE-B2E4-4938B21B22DC

Figs 1–5 , 6–13

#### Diagnosis.

Pronotum with dorsal processes, short and directed dorsally.

#### Description.

Measurements (mm). Total length (from anterior of head to tip of forewings in repose) female 18.6, male 18.4; crown length female 2.9, male 2.8; transocular distance female 4.3, male 4.2; interocular distance female and male 3.0; distance between compound eye and mesal line female and male 1.5; distance between ocellus and mesal line female 0.7, male 0.6; pronotum maximum width female 4.0, male 3.9; pronotum maximum length female and male 3.2; forewing length female 12.1, male 11.8; length of metathoracic femur female 3.0, male 2.6; length of metathoracic tibia female 5.3, male 5.7.

Head (Figs 1–3, 5). Crown maximum length 0.7 times transocular distance and 2.1 times longer than interocular distance in dorsal view; frontoclypeus with deep muscle impressions laterally and planar medially, dorsal surface planar; lateral frontal suture extending onto crown to ocelli. Ocellus located at level of anterior limit of compound eye, distinctly closer to eye than to each other (ratio of distances between eyes vs. between ocelli 2.1). Clypellus anterior margin in lateral view at level of frontoclypeus. Thorax (Figs 1–3, 5). Pronotum maximum width at posterolateral angles 1.1 times wider than transocular distance; maximum length 1.2 times longer than crown length; lateral margins convergent anteriorly, mostly smooth in anterior half, punctate in posterior half; posterior margin transverse; with a pair of suprahumeral processes that are short and directed vertically. Scutellum dorsally smooth, lacking longitudinal carina. Forewing (Figs 1–2, 5) coriaceous; venation with a few extra crossveins between veins R_4+5_ and M_1+2_. Metathoracic leg with femoral setal formula 2:0:0:0 (AD1 and PD1); tibia with anteroventral row (AV) complete with cucullate (*sensu*Deitz 1975) macrosetae; anterodorsal (AD) and posteroventral (PV) rows complete with uniform non-cucullate macrosetae; posterodorsal (PD) row with smaller, more closely spaced, uniform, noncucullate macrosetae; ratio of length of each individual tarsomere by total tarsus length (excluding pretarsus) equal to 0.5, 0.4 and 0.3, respectively. Coloration. Male unicolorus dark brown throughout. Female unicolorous reddish brown throughout.

#### Male terminalia.

**Figures 6–13.**
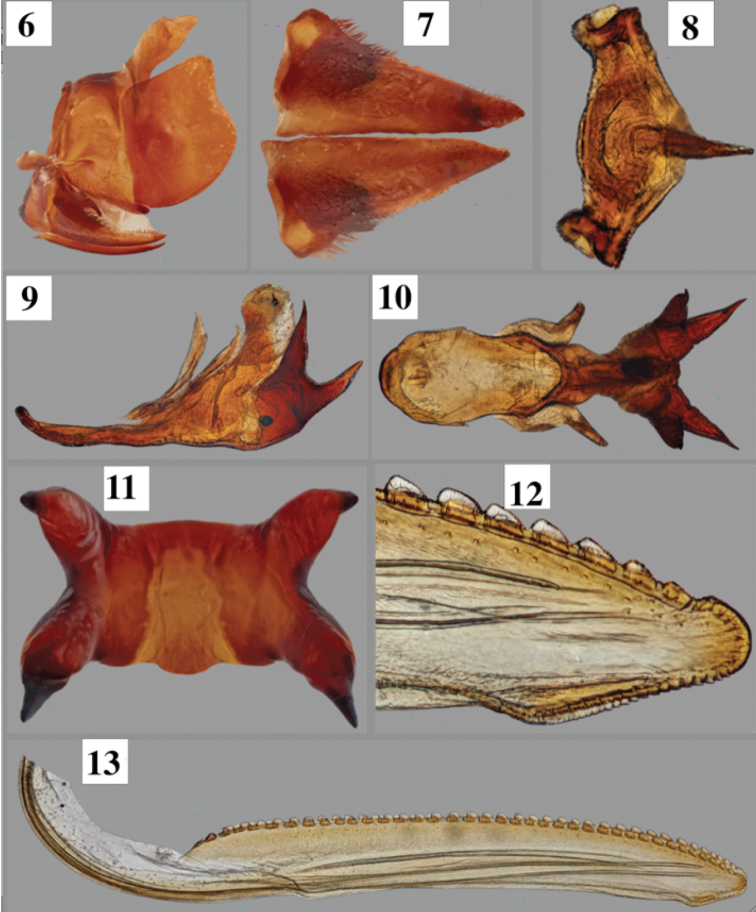
Terminalia of *Pseudopheraheveli***6** pygofer, anal segments, and subgenital plates, lateral view **7** subgenital plates, ventral view **8** male connective, dorsal view **9–11** male aedeagus in lateral, dorsal, and posterior views, respectively **12** detail of apex of female valvula II (posterior row of dentae digitally removed) **13** entire valvula II.

Pygofer (Fig. 6) in lateral view with dorsal margin straight; posterior margin subtruncate. Subgenital plates (Fig. 7) 1.4 times longer than wide at base in ventral view, not fused. Connective (Fig. 8) in dorsal view short (1.5 times wider than long), roughly Y-shaped with anterior arms widely separated and laterally truncate. Style, in dorsal view, without preapical lobe; apex rounded, directed posteriorly beyond connective; ventral margin without preapical dentiform processes. Aedeagus (Figs 9–11) strongly sclerotized, elongate, with 2 pairs of stout spines posteriorly.

#### Female terminalia.

**Figures 14–15.**
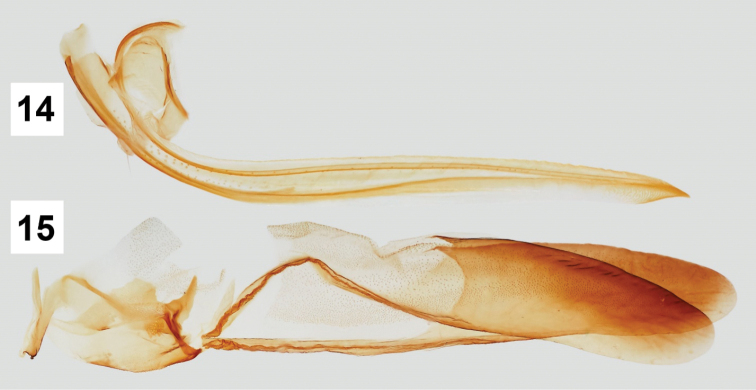
Female valvulae I and III of *Pseudopheraheveli* in lateral views, respectively.

Sternite VII (Fig. 4) transverse, without median emargination of projections; internal sclerotized sternite VIII absent; valvula I (Fig. 14) long, apex acute, lacking spines; valvula II (Figs 12–13) in lateral view serratiform, with 36 teeth, each tooth microserrate on its own dorsal margin; valvula III (Fig. 15) long, broad, apex rounded, basally narrower than distally, lacking spines.

#### Material examined.

1 male “Costa Rica: | Puntarenas Prov. | Monteverde | 12-21 Apr 1984 | S.McKamey, Coll.” (USNM), 1 female: “Monteverde, Costa Rica | Puntarenas Prov. | 1 July 1981 10:00 am | Stuart McKamey Coll. | flying through foliage | 1/2 way up rd. to Reserve” (USNM).

#### Distribution.

Still known only from Monteverde, Costa Rica, inside and just outside the Reserve. Biology and ecology unknown.

#### Notes.

Three species described since Young’s (1968) revision reveal that there is more variation in the shape of the posterior margin of the female sternum than indicated in his generic description. Young (1968) described the female sternum VII of *Pseudophera* as “broadly emarginate medially and with a slight convexity within the emargination,” based on that of *P.divergens* (Schmidt) and presumably also his new species *P.truncata*, of which he had nine females to examine. Similarly, Emmrich’s (1999) illustrations of the female sternum VII indicate that *P.tibialis* Schmidt, *P.contraria* (Walker), *P.heterogena* Schmidt, and *P.paraensis* also have the deep, broad emargination. In contrast, the females of *P.heveli* (Fig. 4), *P.chelicerata* and *P.jimenezi* have the posterior margin of sternum VII transverse, without an emargination or a medial convexity.

## Supplementary Material

XML Treatment for Pseudophera heveli
